# Moderating Role of Creative Mindset in the Effect of Metacognitive Experience on Insight Problem Solving

**DOI:** 10.3390/jintelligence11060099

**Published:** 2023-05-23

**Authors:** Xiaoyu Jia, Ping Li, Qunlin Chen, Weijian Li

**Affiliations:** 1 School of Psychology, Zhejiang Normal University, Jinhua 321000, China; xlxh@zjnu.cn; 2 College of Teacher Education, Southwest University, Chongqing 400715, China; 3 School of Education Science, Guangdong Polytechnic Normal University, Guangzhou 510000, China; 4 Faculty of Psychology, Southwest University, Chongqing 400715, China

**Keywords:** insight problem solving, metacognitive experience, processing fluency, creative mindset, Chinese logogriphs, font style

## Abstract

Metacognitive experience, measured by processing fluency, contributes to divergent thinking performance; however, whether it exhibits varying effects on insight problem-solving remains unknown. Additionally, as individuals’ interpretation of metacognitive experience is influenced by their creative mindset, whether creative mindset plays a role in the relationship between metacognitive experience and insight problem-solving is another issue. In Experiment 1, a Chinese logogriph task was used to investigate insight problem-solving performance. The font style of logogriphs (easy versus difficult) was used to alter the ease of processing. The results showed that individuals had lower performance accuracy for logogriphs presented in difficult font styles, suggesting the negative effect of metacognitive disfluency experience on logogriph solving. In Experiment 2, different creative mindsets (entity versus incremental) were activated in individuals via prime manipulation. Individuals with an incremental creative mindset had a significantly higher performance accuracy and longer reaction time for logogriphs presented in difficult font styles than individuals with an entity creative mindset, suggesting that an incremental creative mindset might counteract the negative effect of metacognitive disfluency experience on logogriphs solving. These findings suggest that metacognitive disfluency experience has a negative effect on insight problem-solving and that a creative mindset moderated this effect.

## 1. Introduction

Creativity, which has been defined as the ability to produce products that possess both novelty and utility (Sternberg and Lubart 1991), is an interactive system involving creative thinking, personality, and environment (Jausovec 1994). Over the past few decades, many studies have supported the idea of the top-down regulation of metacognition, in which individuals consciously monitor and control their cognitive activities (Flavell 1976), which influences creativity (Berkowitz and Ansari 2008; Erbas and Bas 2015; Jia et al. 2022; Lizarraga and Baquedano 2013; Preiss et al. 2016). Of note, the important role of a sub-component of metacognition—metacognitive experience—which indicates individuals’ subjective perception regarding the ease or difficulty of certain cognitive operations in creativity, has generated particular interest (Jia et al. 2019; Mehta et al. 2012; Puente-Díaz 2023; Threadgold et al. 2019).

Generally, the metacognitive experience can be indicated by a metacognitive cue of processing fluency (Koriat et al. 2004). According to the ease-of-processing hypothesis (Koriat 2008), individuals’ metacognitive experience influences their goal setting (Storbeck and Clore 2007), work effort (Miele and Molden 2010), strategy choice (Lucas and Nordgren 2015), and processing style (Alter et al. 2007) during cognitive activities, all of which play key roles in creativity (Gilhooly et al. 2007). Eysenck (1993) suggests that creativity is a constant oscillation between divergent and convergent thinking. Specifically, divergent thinking refers to the expansive generation of novel ideas for an open-ended problem, such as the classic Alternative Uses Task, whereas convergent thinking emphasizes the production of a single response from all possible answers to a given question (Guilford 1967), such as insight problem-solving tasks (Luo and Knoblich 2007). It has been confirmed that these two different kinds of creative tasks have different cognitive processing mechanisms (Benedek et al. 2011; Fink et al. 2012). Few studies have found that metacognitive disfluency experience (i.e., experiencing much more difficulty in processing) is positively related to divergent-thinking task performance (Mehta et al. 2012). Moreover, previous studies have suggested that individuals with different types of creative mindsets, namely whether they view creativity as an entity or incremental manner (Oconnor et al. 2013), exhibit significant differences in their interpretation of the metacognitive experience indicated by processing fluency (Miele et al. 2011) and subsequent cognitive behaviors (Labroo and Kim 2009; Miele et al. 2011; Miele and Molden 2010; Thomas and Morwitz 2008). Therefore, this study investigated whether metacognitive disfluency experience plays a different role in insight problem-solving tasks. Additionally, we investigated the role of an individual’s creative mindset in the relationship between metacognitive experience and insight problem-solving.

### 1.1. Metacognitive Experience and Creativity

Accumulating evidence indicates the relationship between creativity and metacognitive experience, which is reflected by processing fluency (Lucas and Nordgren 2015; Mehta et al. 2012; Threadgold et al. 2019). Mehta et al. (2012) revealed that participants completed the brick uses task either shortly after background noise (low vs. moderate volume) began to play or after a delay. Subsequently, a seven-point scale containing three questions (e.g., “How distracting did you find the room ambiance while completing the study?”) was used to assess their fluency of metacognitive experience induced by various noise levels. The result showed that moderate (vs. low) levels of noise-induced greater metacognitive disfluency experience and subsequently enhanced the generation of creative ideas regardless of the noise timing, suggesting that the metacognitive disfluency experience contributes to divergent thinking task performances.

Different degrees of metacognitive fluency experience can induce different kinds of processing styles (Alter et al. 2007); metacognitive fluency experience induces a more intuitive processing style (System 1), whereas metacognitive disfluency experience induces a more analytical processing style (System 2). Evidence from brain-imaging studies has supported the notion that metacognitive disfluency experience activates parts of the anterior cingulate and prefrontal cortexes (Boksman et al. 2005), thereby allowing people to think carefully and use analytical processing (Kühl et al. 2014) to complete creative tasks. Some studies have suggested that individuals’ cognitive persistence and effort from system two played a positive role in creativity (Lucas and Nordgren 2015; Nijstad et al. 2010; Rubenstein et al. 2019). Other studies, however, argued that unconscious awareness or intuitive insight contributed to creativity (Ansburg and Hill 2003; Chen et al. 2012). The general agreement of different dual-process models of creativity, such as the Dual Pathway Model (Nijstad et al. 2010) and the Dual State Model (Howardjones 2002), is that creativity may most likely result from the joint operation of both automatic and analytical processes.

Previous studies about the relationship between metacognitive experience and creativity have experienced some limitations. First, individuals’ metacognitive fluency experiences were aroused by indirect operations, such as noise or background music (Mehta et al. 2012; Threadgold et al. 2019); thus, more direct ways of disrupting people’s processing fluency could be used in the future. Second, because divergent and convergent thinking tasks have different cognitive processing mechanisms (Benedek et al. 2011; Fink et al. 2012), whether metacognitive disfluency experience has different roles in convergent thinking tasks, such as insights into problem-solving, should be clarified. To address these limitations, this study used the Chinese logogriph task, which is traditionally classified as an insight problem involving an Aha experience of generating a solution (Metcalfe and Wiebe 1987; Qiu et al. 2010). Specifically, Chinese logogriphs comprise puzzles and their answers. A puzzle might be a Chinese phrase or proverb, while the corresponding answer is a Chinese character. In the process of Chinese logogriph solving, people should first search for the deep semantic meanings of the puzzles along with the mental sets breaking and then form novel associations by recombining the structural components of puzzles (Luo et al. 2011). The solution of logogriphs is unique and can be easily scored to reflect an individual’s creative ability (Sprugnoli et al. 2017). In addition, font style manipulation, in which words are presented in either an easy font (e.g., Times New Roman) or a difficult font (e.g., a small gray, italicized font), has been confirmed to be effective in inducing different levels of processing fluency (Alter and Oppenheimer 2009; Alter et al. 2007; Jia et al. 2016; Novemsky et al. 2007; West and Bruckmüller 2013). The main idea is that words presented in a difficult font could disrupt participants’ subjective feeling of fluency by affecting experienced readability (Reber et al. 2004). Empirically, Li et al. (2016) manipulated the words either in Imitation Song (an easy font style) or Teng Cheung (a difficult font style) and found that the effect of animacy on metamemory decreased or disappeared in the processing disfluency condition induced by presenting words in a difficult font style. In this study, to investigate the role of metacognitive experience in the processing disfluency condition, we used a similar font style manipulation to alter the ease with which Chinese logogriphs could be read.

Aiello et al. (2012) conducted a study in which participants were asked to complete the remote association task (RAT) before or after an artificial grammar task. In some conditions, an instructor instructed them to “use your gut” and rely on their intuitive decision-making. The results revealed that participants who performed the artificial grammar task with the “use your gut” instruction before the RAT task showed improved performance in the RAT task. This suggests that adopting a less analytical approach, as encouraged by the “use your gut” instruction, benefited convergent thinking performance. As a type of convergent thinking task, Chinese logogriph solving also relies on insight, wherein the solution occurs in a sudden and unpredictable manner with little or no conscious processing (Metcalfe and Wiebe 1987; Qiu et al. 2010), whereas excessive analytical processing or purposeful thinking might lead to creative impasses, and thus, the inhibition of the logogriphs solving performance. As Topolinski and Reber (2010) indicated, the solutions to problems produced by insight other than analysis are a consequence of high fluency processing, and we propose that a negative effect of the metacognitive disfluency experience on Chinese logogriph performance would be expected, as metacognitive disfluency experience may activate individuals much more analytical processing to inhabit logogriphs solving.

### 1.2. Metacognitive Experience, Creative Mindset, and Creativity

Potentially, the effect of metacognitive experience on creative thinking might be moderated by individuals’ domain-specific implicit theories of creativity (i.e., creative mindsets; Oconnor et al. (2013)). Generally, creative mindsets can be divided into two types: entity (viewing creativity as stable and unable to change) and incremental (viewing creativity as malleable and able to grow or decline; Andiliou and Murphy (2010)). Individuals with these different creative mindsets have different cognitive processing characteristics and thus perform differently in creative tasks (Benedek et al. 2011; De Dreu et al. 2008; Roskes et al. 2012). For example, Oconnor et al. (2013) found that creative mindsets measured by both a five-item Likert scale and prime operations using a series of quotations were significantly related to a series of creativity measures, such as the self-perceptions of creativity, lifetime creative achievement, and unusual uses task performance. Consistently, Karwowski (2014) developed a creative mindset measuring instrument (i.e., Creative Mindset Scale, CMS) and found a similar association between creative mindsets and creative self-efficacy, as well as a creative personal identity and insight problem-solving efficiency.

Moreover, a fair amount of work indicated that individuals’ implicit beliefs could influence their interpretation of metacognitive experience reflected by processing fluency and the cognitive behaviors that follow (Labroo and Kim 2009; Miele et al. 2011; Miele and Molden 2010; Thomas and Morwitz 2008). Specifically, individuals with an incremental belief might interpret metacognitive disfluency experience as a lack of effort and thus would show more cognitive persistence to compensate for the performance deficit. In contrast, individuals with an entity belief might interpret metacognitive disfluency as a deficiency in their ability and, thus, were more likely to give up the task. Evidence for this moderating effect of implicit beliefs on the relationship between metacognitive experience and task performance was obtained from studies on the judgment of learning (Molden and Dweck 2006), reading comprehension (Miele and Molden 2010), and learning achievement (Blackwell et al. 2007). An important question is whether there is such an effect in the context of insight problem-solving. To answer this question, we used a multiple-choice task, which was similar to that used by Oconnor et al. (2013), to prime creative mindsets (entity vs. incremental) among the participants. Moreover, self-paced study time, as a spontaneous and naturalistic indicator of self-generated effort and cognitive persistence (Koriat and Ma’ayan 2005; Miele and Molden 2010), was used to investigate how much effort participants with different kinds of creative mindsets could put into solving logogriphs. The total amount of reaction time was recorded in this study.

Overall, the current study investigated the: (a) influence of metacognitive experience reflected by processing fluency on Chinese logogriph problem-solving and (b) the role of individual creative mindset on the relationship between metacognitive experience and Chinese logogriph problem-solving. Based on the observations of previous studies, we developed the following hypothesis:

**Hypothesis 1:**  *There is a negative effect of metacognitive experience on Chinese logogriph performance. Individuals’ metacognitive disfluency experience may inhibit their logogriphs solving.*

**Hypothesis 2:**  *Individual creative mindsets play a moderating role in the relationship between metacognitive experience and Chinese logogriph problem-solving. Compared with individuals that have an entity creative mindset, those with an incremental mindset show much more cognitive persistence to compensate for the performance deficit of metacognitive disfluency experience in logogriphs solving.*

## 2. Experiment 1

In Experiment 1, font style manipulation was applied to manipulate participants’ processing fluency during the Chinese logogriph task. After completing the puzzles, participants were asked to evaluate the emotional experience of their insight judgment to reflect their extent of solving the puzzles with heuristic and insight processing. We expected that puzzles presented in the difficult font style would disrupt participants’ subjective feeling of fluency (Alter and Oppenheimer 2009; Jia et al. 2016; Li et al. 2016), which, in turn, would weaken their logogriphs solving. In addition, because item difficulty, an important attribute of the Chinese logogriph, can strongly influence people’s cognitive resource allocation and effort-making in the process of task completion (Qiu et al. 2008), we examined whether metacognitive experience continued to influence logogriph solving when this variability was presented.

### 2.1. Methods

#### 2.1.1. Participants

We used G*Power 3 software (Faul et al. 2007) to determine the minimum sample with an effect size of 0.25, an alpha of 0.05, and a power of 0.95. The expected sample size was 36. Fifty-five university students participated in this study with compensation. One participant did not complete the study and was thus excluded from further analyses. None of the 54 included participants (21 males, *M* = 20.59, *SD* = 1.59) had participated in other similar studies.

#### 2.1.2. Design

The experiment was evaluated as having a 2 (task difficulty: easy or difficult) × 2 (font style: easy or difficult) within-subject design. The dependent variables were the mean proportion of logogriphs correctly answered (performance accuracy) and its insight judgment value (insight value).

#### 2.1.3. Chinese Logogriphs Materials

From the Chinese logogriphs pool developed by Wu et al. (2009), we selected 40 logogriphs that were evaluated as being highly interesting (mean scores > 3.5) by 30 independent subjects on a 5-point scale ranging from “1 = very boring” to “5 = very interesting” in a pretest. Approximately one-half of the 40 logogriphs were difficult, while the other one-half was easy. The difficulty of the logogriphs was calculated by the following formula:

$$\mathrm{The}\;\mathrm{difficulty}\;\mathrm{of}\;\mathrm{logogriph}=1-\frac{The\;number\;of\;people\;who\;answered\;the\;logogriphs\;correctly}{The\;total\;number\;of\;individuals\;assessed}$$

We found significant differences in the degree of difficulty between easy logogriphs (e.g., the answer to the puzzle “昨日告别”, which means “say goodbye to yesterday”, is “乍”, which literally means “suddenly”) and difficult logogriphs (e.g., the answer to the puzzle “欢迎光临”, which means ”welcome to …”, is “闪”, which literally means “flash”), *t*(19) = −18.23, *p* < 0.001, *d_z_* = 4.56. The length of most logogriphs was between 2 and 5 characters, and each answer was unique and a single character.

#### 2.1.4. Font Style Manipulation

The font style manipulation was similar to that conducted by Jia et al. (2016) and Li et al. (2016). Specifically, logogriphs in the easy font style condition were presented in Imitation Song, a bold font, such as “小人国” (Lilliput), while logogriphs in the difficult font style condition were presented in Teng Cheung, a similar bold font that is also italicized, such as “
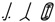
” (Lilliput). In a pretest, we recruited 30 independent raters to evaluate the processing fluency of these font styles. They were each shown a sample logogriph and asked to rate it on a five-point scale ranging from “1 = very easy to read” to “5 = very difficult to read.” The results showed that the logogriphs presented in the easy font style were much easier to read (*M* = 1.05, *SD* = 0.22) than those presented in a difficult font style (*M* = 2.95, *SD* = 0.74), *t*(29) = −12.46, *p* < 0.001, *d_z_* = 2.71.

Furthermore, each of the two difficult sets of logogriphs was then randomly divided into two subsets of 10 logogriphs. One subset was presented in the easy font style, and the other was presented in the difficult font style. Two additional logogriphs (one for each font style) were presented randomly at the beginning of the list for practice. Participants’ answers for these logogriphs were excluded from the final analyses. The difficulty of the four kinds of logogriphs is presented in Table 1.

#### 2.1.5. Procedure

Each participant was tested individually on a computer. First, a puzzle was present in the center of the screen for 10 s. Participants were instructed to try and solve the puzzle and provide their answers by pressing a key. More specifically, participants were required to press “1” on the keyboard as soon as they arrived at the answer. After they answered, they were required to evaluate the emotional experience of their insight judgment on a five-point scale ranging from “have no emotional experience” to “have the strongest emotional experience.” If participants arrived at no answer, they were asked not to press any key, and the next puzzle would appear automatically once 10 s passed. It is worth mentioning that four types of puzzles were presented in random forms throughout the experiment.

#### 2.1.6. Manipulation Check

After the Chinese logogriph task, we evaluated the effectiveness of the processing fluency manipulation. Specifically, participants were asked to judge their degree of processing fluency when processing two kinds of font styles on a 5-point scale ranging from “1 = high disfluency” to “5 = high fluency”.

### 2.2. Results

#### 2.2.1. Manipulation Check

To evaluate the effectiveness of the processing fluency manipulation, a paired-sample *t*-test was conducted on processing fluency. The results indicated that the easy font style led to a significantly greater processing fluency (*M* = 4.13, *SD* = 0.93) than the difficult font style (*M* = 2.54, *SD* = 0.94), *t*(53) = 9.97, *p* < 0.001, *d*_z_ = 1.35, indicating that the processing fluency manipulation was effective.

#### 2.2.2. Task Performance

**Figure 1:**
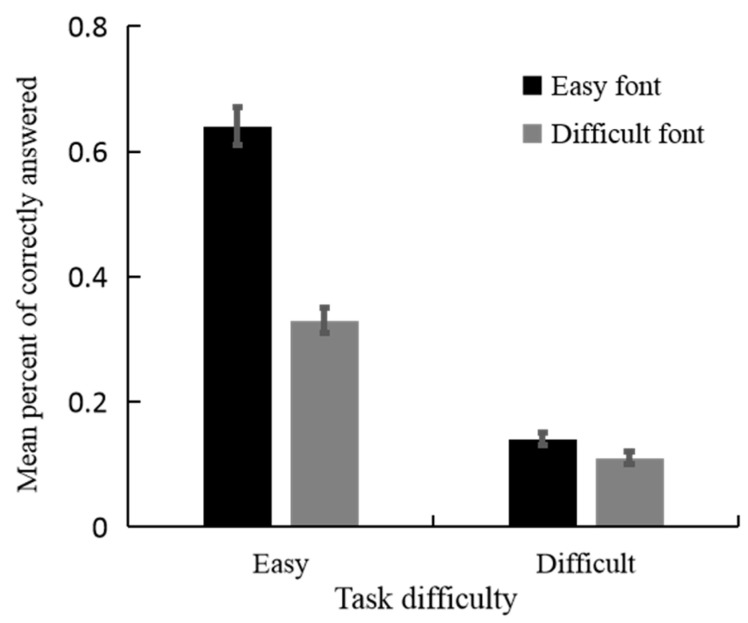
Task performance by task difficulty and font style in experiment 1. Error bars: 95% confidence intervals.

We analyzed the effect of task difficulty and font style on performance accuracy using a linear mixed-effects model with the lmer package in R (Bates et al. 2015). In the mixed-effect model, participants were included as random variables. Therefore, fixed and random effects could be estimated in one single analysis, which offered additional advantages over the traditional repeated-measures analysis of variance (Jaeger 2008). Estimates of the fixed effects are presented in Table 2. First, we observed the significant main effect of task difficulty (*β* = −0.5, *t* = −20.99, *p* < 0.001), indicating that the performance accuracy was higher for easy logogriphs (*M* = 0.48, *SD* = 0.02) than for difficult logogriphs (*M* = 0.12, *SD* = 0.01). Second, the main effect of the font style was significant (*β* = −0.31, *t* = −13.03, *p* < 0.001), indicating that the performance accuracy was higher for logogriphs presented in the easy font style (*M* = 0.39, *SD* = 0.02) than for those presented in the difficult font style (*M* = 0.22, *SD* = 0.02). There was a significant two-way interaction between task difficulty and font style (Figure 1; *β* = 0.27, *t* = 8.17, *p* < 0.001). Specifically, the performance accuracy of easy logogriph was significantly higher than that of difficult logogriphs for both easy font styles (easy logographs: *M* = 0.64, *SD* = 0.20; difficult logogriphs: *M* = 0.14, *SD* = 0.08; *β* = 0.5, *t* = 20.99, *p* < 0.001) and difficult font styles (easy logographs: *M* = 0.33, *SD* = 0.18; difficult logogriphs: *M* = 0.11, *SD* = 0.09; *β* = 0.22, *t* = 9.44, *p* < 0.001). For easy logogriphs, the performance accuracy in the easy font style (*M* = 0.64, *SD* = 0.20) was significantly higher than that of the difficult font style (*M* = 0.33, *SD* = 0.18), *β* = 0.31, *t* = 13.03, *p* < 0.001, whereas for difficult logogriphs, there was no significant difference in the performance accuracy between the easy font style (*M* = 0.14, *SD* = 0.08) and difficult font style (*M* = 0.11, *SD* = 0.09), *β* = 0.03, *t* = 1.48, *p* = 0.14.

#### 2.2.3. Insight Value

**Figure 2:**
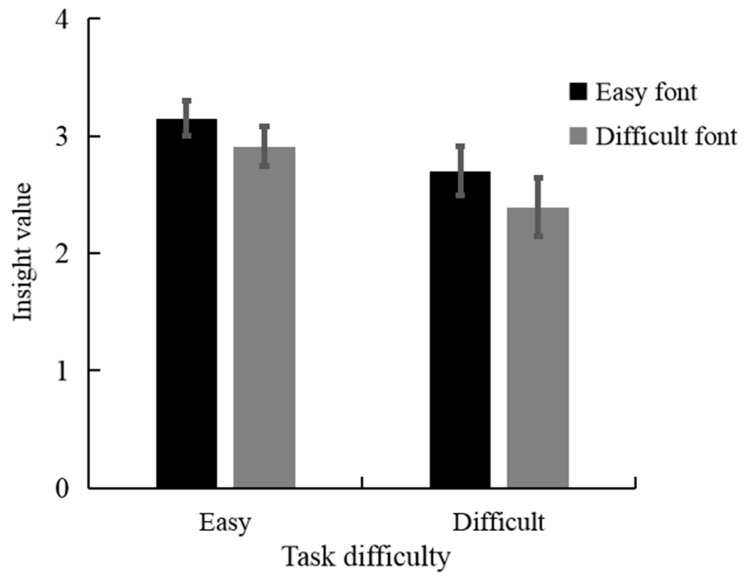
Insight value by task difficulty and font style in experiment 1.

We analyzed the effect of task difficulty and font style on the insight value using a linear mixed-effects model. Estimates of the fixed effects are presented in Table 2. There was a significant main effect of task difficulty (*β* = −0.50, *t* = −2.22, *p* < 0.05), indicating that participants had higher insight values for easy logogriphs (*M* = 3.03, *SD* = 0.15) compared to difficult ones (*M* = 2.54, *SD* = 0.18). However, the main effect of the font style was not significant (*β* = −0.24, *t* = −1.18, *p* = 0.24). Moreover, the interaction between task difficulty and font style was not significant (Figure 2, *β* = −0.07, *t* = −0.26, *p* = 0.80).

### 2.3. Discussion

Experiment 1 revealed that both font style and task difficulty affected the logogriphs-solving performance. The finding that task difficulty affected creative problem-solving was consistent with that of a previous study (Threadgold et al. 2019). In addition, individuals had a higher insight value and performance accuracy under the processing fluency experience triggered by an easy font style, indicating that fast and unconscious processing might promote logogriphs solving (Ansburg and Hill 2003; Chen et al. 2012).

## 3. Experiment 2

Experiment 1 showed that the metacognitive disfluency experience had a negative effect on Chinese logogriphs solving. In Experiment 2, we investigated the role of individuals’ creative mindsets in this relationship in an experimental setting. A multiple-choice task, as described by Oconnor et al. (2013), was used to prime the creative mindsets (entity vs. incremental) among participants. We hypothesized that individuals with an incremental creative mindset would correctly solve a significantly greater proportion of logogriphs than individuals with an entity mindset under the metacognitive disfluency experience condition. If creative mindset plays a moderating role in the relationship between metacognitive experience and Chinese logogriphs solving, this relationship would disappear among participants primed for an incremental creative mindset but not among those primed for an entity creative mindset. Only medium-difficulty Chinese logogriphs were used to avoid the confusing effect of logogriphs difficulty on their solving performance in this experiment.

### 3.1. Methods

#### 3.1.1. Participants

G*Power 3.1 was used to determine the minimum sample size with an effect size of 0.25, an alpha of 0.05, and a power of 0.95. The expected sample size was 54. In total, 80 university students participated in this study with compensation. Two participants did not complete the study and were, thus, excluded from further analyses. None of the 78 effective participants (18 males; *M* = 20.83, *SD* = 1.72) had previously participated in Experiment 1.

#### 3.1.2. Design

The experiment used a 2 (creative mindset: entity or incremental) × 2 (font style: easy or difficult) mixed design. The font style, similar to Experiment 1, was a within-subject variable, while creative mindset was a between-subject variable. The dependent variables were the mean proportion of logogriphs correctly answered (performance accuracy) and the reaction time on the Chinese logogriph task.

#### 3.1.3. Chinese Logogriphs Materials

Thirty-two medium-difficulty Chinese logogriphs based on the study by Wu et al. (2009) were chosen, such as the answer to the puzzle “两人走钢丝”, which means “two men walk on a tightrope”, is “丛”, which literally means “clump”.

#### 3.1.4. Font Style Manipulation

The font style manipulation was similar to that in Experiment 1. The 32 medium-difficulty Chinese logogriphs were randomly and evenly divided into two sets: one was presented in the easy font style and the other in the difficult font style. Four additional logogriphs were chosen for practice but were excluded from all the analyses.

#### 3.1.5. Creative Mindset Manipulation

To prime the creative mindsets of participants, we used a multiple-choice task similar to that of Oconnor et al. (2013). In this task, participants saw eight ostensibly accurate quotations, and they had to select the author of the quotations among three options. Each of the eight quotations was presented in 20 s, and the correct answer was provided for 5 s once participants had made a choice. The first six quotations did not relate to creativity, while the final two quotations reflected different creative mindsets. For the entity manipulation condition, these two quotations described how creativity is inherited, fixed, and unchangeable. In contrast, for the incremental manipulation condition, the two quotations described that creativity is malleable and changeable. The accuracy of this task was not relevant to the purpose of the study, so we did not analyze this.

#### 3.1.6. Procedure

Each participant was tested individually on a computer. They were told that they would first complete a multiple-choice task and then a Chinese logogriph task. The Chinese logogriph task had the same procedure as Experiment 1, with two exceptions: (1) each puzzle was presented for 30 s, rather than 10 s, in the initial phase to give participants sufficient time to search for an answer. Participants were told that they could study each puzzle at their own pace for a maximum of 30 s. Their self-paced study time was recorded. (2) Insight judgment was removed to shorten the Chinese logogriph task time. This may be effective for ensuring the validity and durability of initial creative mindset priming.

#### 3.1.7. Manipulation Checks

After both tasks, we evaluated the effectiveness of the processing fluency manipulation and the creative mindset manipulation. The processing fluency manipulation check was the same as in Experiment 1. The creative mindset manipulation check utilized two methods to ensure its effectiveness. First, participants were asked, “Which of the following two statements about creativity do you agree with?” They had a choice of two options (A: An individual’s creativity is inherently stable and hard to improve through hard work; B: An individual’s creativity is malleable and can be improved through hard work). Second, participants were asked to complete a three-item implicit theory of the creativity scale adapted from that by Hong et al. (1999). The items were “My creativity level is certain, and I really cannot do much to change it”, “I think creativity is an ability that is unlikely to change”, and “I can learn new knowledge, but I cannot really improve my creativity.” Higher scores indicated a greater belief in the entity theory of creativity.

### 3.2. Results

#### 3.2.1. Manipulation Checks

A paired-sample *t*-test for processing fluency was computed to examine the effectiveness of the processing fluency manipulation. The results showed that the easy font style led to a significantly greater processing fluency (*M* = 3.49, *SD* = 1.17) than the difficult font style (*M* = 2.47, *SD* = 0.92), *t*(77) = 6.85, *p* < 0.001, *d_z_* = 0.77, suggesting that the processing fluency manipulation was effective.

An independent-sample t-test was conducted to examine the difference in scores on the implicit theory of the creativity scale between participants primed for an entity mindset and those primed for an incremental mindset to examine the effectiveness of the creative mindset manipulation. The results indicated that participants primed for the entity mindset (*M* = 8.26, *SD* = 0.49) scored higher than those primed for the incremental mindset (*M* = 7.20, *SD* = 0.32), *t*(76) = 1.79, *p* < 0.05, *d*_z_ = 2.56. Additionally, a chi-square test was conducted for participants’ answers to the question about the nature of creativity according to their creative mindset priming. The consistent number, which indicates the prime condition consisting of the category based on the implicit theory of the creativity scale, was computed. For example, if a person was not only primed for the incremental mindset but also categorized with an incremental belief based on his scores on the implicit theory of the creativity scale, this person was classified as having a consistent number for the incremental mindset group. Accordingly, the consistent number was 35 and 27 in the incremental and entity mindset prime conditions, respectively. The results showed that the proportions of consistent and inconsistent numbers differed significantly according to the creative mindset groups (χ^2^ = 26.37, *p* < 0.001). These results indicated that the creative mindset manipulation was effective.

#### 3.2.2. Task Performance

**Figure 3:**
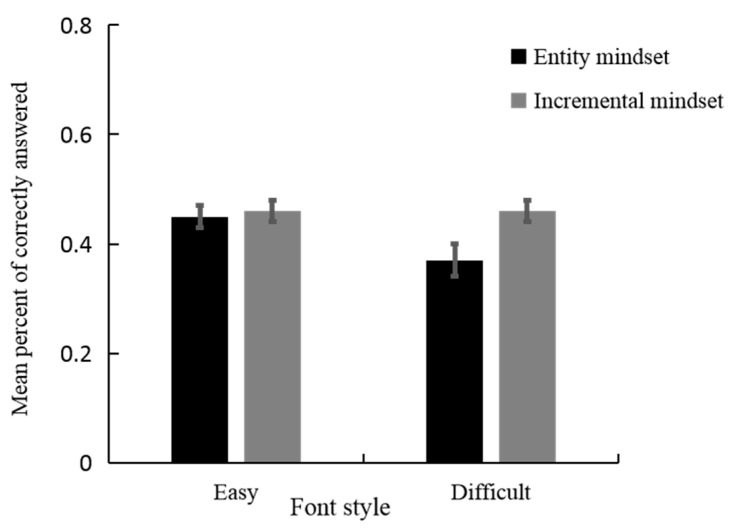
Task performance by creative mindset and font style in experiment 2.

We analyzed the effect of creative mindset and font style on performance accuracy using a linear mixed-effects model. Estimates of the fixed effects are presented in Table 3. There was a significant main effect on the font style (*β* = −0.08, *t* = −3.22, *p* < 0.005), indicating that the performance accuracy was higher for logogriphs presented in an easier font style (*M* = 0.46, *SD* = 0.02) than those presented in a difficult font style (*M* = 0.41, *SD* = 0.02). The main effect of a creative mindset was not significant (*β* = 0.02, *t* = 0.47, *p* = 0.64). There was a significant two-way interaction between the creative mindset and font style (Figure 3, *β* = 0.08, *t* = 2.27, *p* < 0.05). Specifically, for logogriphs presented in a difficult font style, the performance accuracy of individuals primed for the incremental mindset (*M* = 0.46, *SD* = 0.13) was significantly higher than that for the entity mindset (*M* = 0.37, *SD* = 0.17), *β* = −0.10, *t* = −2.90, *p* < 0.005, whereas for logogriphs presented in the easy font style, there was no significant difference between the two groups(incremental condition: *M* = 0.46, *SD* = 0.15; entity condition: *M* = 0.45, *SD* = 0.13; *β* = −0.02, *t* = −0.47, *p* = 0.64). In addition, the performance accuracy was higher for logogriphs presented in an easier font style (*M* = 0.45, *SD* = 0.13) than for those presented in a difficult font style (*M* = 0.37, *SD* = 0.17) among the participants primed for the entity mindset, *β* = 0.08, *t* = 3.22, *p* < 0.005. However, there was no significant difference between the easy and difficult font style conditions among participants primed for the incremental mindset (easy font style: *M* = 0.46, *SD* = 0.15; difficult font style: *M* = 0.46, *SD* = 0.13; *β* = 0.00, *t* = 0.01, *p* = 0.99).

#### 3.2.3. Reaction Time

**Figure 4:**
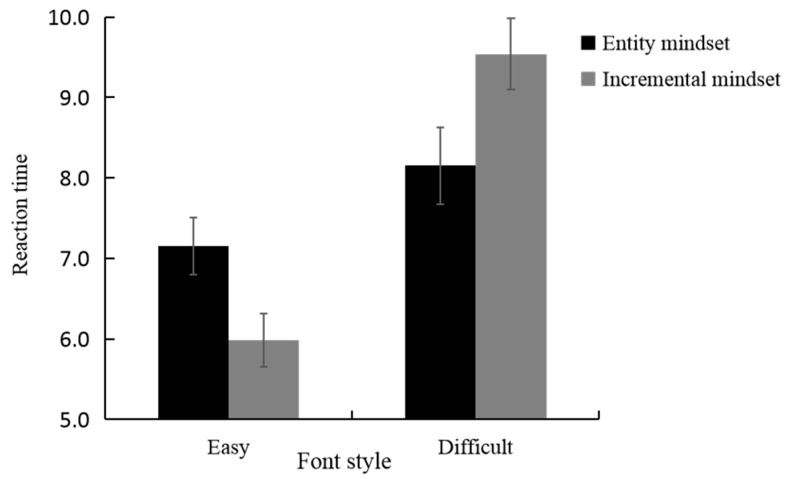
Reaction time by creative mindset and font style in experiment 2.

We analyzed the effect of a creative mindset and font style on reaction time using a linear mixed-effects model. Estimates of the fixed effects are presented in Table 3. There was a marginal main effect on the font style (*β* = 1.00, *t* = 1.73, *p* = 0.09), indicating that the reaction time was longer for logogriphs presented in a difficult font style (*M* = 8.84, *SD* = 0.33) than for those in an easy font style (*M* = 6.56, *SD* = 0.24). There was a significant main effect of the creative mindset (*β* = −1.17, *t* = −2.03, *p* < 0.05), indicating that the reaction time was longer for individuals primed for the incremental mindset (*M* = 7.76, *SD* = 0.28) than for those primed for the entity mindset (*M* = 7.64, *SD* = 0.28). There was a significant two-way interaction between the creative mindset and font style (Figure 4, *β* = 2.57, *t* = 3.15, *p* < 0.005). Specifically, for logogriphs presented in an easy font style, the reaction time of individuals primed for the incremental mindset (*M* = 5.98, *SD* = 0.33) was significantly shorter than that of individuals primed for the entity mindset (*M* = 7.15, *SD* = 0.35), *β* = 1.17, *t* = 2.03, *p* < 0.05. However, for logogriphs presented in a difficult font style, the reaction time of individuals primed for the incremental mindset (*M* = 9.54, *SD* = 0.44) was significantly longer than that of individuals primed for the entity mindset (*M* = 8.15, *SD* = 0.48), *β* = −1.40, *t* = −2.42, *p* < 0.05. Additionally, the reaction time of logogriphs presented in the difficult font style was longer than that of the easy font style among individuals primed for the incremental mindset (difficult font style: *M* = 9.54, *SD* = 0.44; easy font style: *M* = 5.98, *SD* = 0.33; *β* = −3.57, *t* = −6.18, *p* < 0.001) and those primed for the entity mindset (difficult font style: *M* = 8.15, *SD* = 0.48; easy font style: *M* = 7.15, *SD* = 0.35; *β* = −0.99, *t* = −1.73, *p* = 0.08).

### 3.3. Discussion

Experiment 2 found that priming individuals’ incremental creative mindset improved their response time and task accuracy under the condition of logogriphs presented in the difficult font style, indicating that creative mindset played a moderating role in the influence of metacognitive disfluency experience on logogriphs solving. The types of creative mindsets that an individual owned affected their interpretation of the metacognitive experience (Thomas and Morwitz 2008; Miele et al. 2011). The metacognitive disfluency experience made individuals with an incremental creative mindset devote a much greater cognitive effort to search for solutions in the logogriphs problem space.

## 4. Discussion

In the present study, we investigated the relationship among metacognitive experience, creative mindset, and insight problem-solving. The results found that individuals had fewer correct answers for logogriphs presented in the difficult font style, suggesting that metacognitive disfluency experience had a negative effect on Chinese logogriphs solving. Moreover, individuals with different types of creative mindsets performed differently on the Chinese logogriph task under varying degrees of metacognitive fluency experience. Compared with individuals that had an entity mindset, individuals with an incremental mindset spent more time on and correctly solved a significantly higher proportion of logogriphs presented in the difficult font style. Perhaps the cognitive persistence associated with the latter creative mindset helped to mitigate the negative effect of metacognitive disfluency experience on Chinese logogriphs solving. That is, individuals’ creative mindsets, primed by a multiple-choice task, moderated the relationship between metacognitive experience and Chinese logogriphs solving ability.

Dual-process theories indicate that problem-solving utilizes two distinct processing systems: a quick, intuitive, and unconscious system (system 1) and a slow, analytic, and conscious system (system 2) (James 2007). The use of a specific processing system is based on the subjective experience of processing fluency (Alter et al. 2007; Botvinick et al. 2001; Lieberman et al. 2002). That is, if the information is processed easily, system 1 is triggered. However, if the information is difficult to process, system 2 is triggered. The result of experiment 1 suggested that the intuitive process style triggered by the metacognitive fluency experience contributed to their performance on the Chinese logogriph task. This is in line with the results of previous research (Aiello et al. 2012). The metacognitive fluency experience induced by an easy font style can more likely influence individuals to use insights to solve logogriphs. Processing fluency elicited a positive effect, which was an important component of the Aha experience (Skaar and Reber 2020). However, notably, we did not find any difference in the insight judgment value between the two processing fluency conditions. A more accurate explanation of the Aha experience instruction should be provided to participants prior to the experiment (Jungbeeman et al. 2004).

Since Chiu et al. (1997) introduced the importance of implicit theories of creativity (creative mindsets) for creative performance, related empirical studies have added supporting evidence (Karwowski 2014; Oconnor et al. 2013). Moreover, numerous studies have suggested that individuals with different implicit theories might interpret processing disfluency as either a lack of effort or an ability deficiency, which could further influence task-relevant processes such as cognitive persistence, strategy selection, learning styles, and, ultimately, the final task performance (Miele et al. 2011, 2013; Miele and Molden 2010; Molden and Dweck 2006). In experiment 2, we found that compared with individuals with an entity creative mindset, individuals with an incremental creative mindset appeared to show more cognitive persistence, possibly to compensate for the negative effect of processing disfluency on logogriphs solving.

According to the dual pathway model of creativity (De Dreu et al. 2008), creative insight problems can be solved via heuristic, effortless processing (Brand et al. 2007) or cognitive persistence (i.e., prolonged analytical processing on the task). Sowden et al. (2015) summarized several different dual-processing models of creativity, such as the aforementioned Dual Pathway Model (Nijstad et al. 2010), Dual State Model (Howardjones 2002), and Honing Theory (Gabora 2005), wherein the general agreement was that creativity might most likely result from the joint operation of both automatic and analytical processes. The most notable problem was how the extent and timing of shifting between these two processes varied among individuals. Previous studies have suggested that the Chinese logogriph task can be solved using both insight or analytical problem-solving processes (Sprugnoli et al. 2017) and that this strategy shifting may more likely serve the purpose of engaging in the solving process, which is inconsistent with classic insight problems such as the Nine-dot problem and Dunker candle task. That is, Chinese logogriphs presented in the difficult font style might activate analytic reasoning (Alter et al. 2007), leading to a creative impasse and uncertainty about how to proceed (Förster et al. 2004). However, individuals with an increment mindset might interpret their metacognitive disfluency as a lack of effort on their part, prompting them to allocate more attention and enhance their motivation and cognitive perseverance to tasks. Moreover, they may adopt a search strategy (Kounios et al. 2008) to systematically evaluate the starting- and goal states of the problem, seeking out a number of possible paths with which to discover the solution, which is relatively effort- and time-consuming but ultimately beneficial to solving logogriphs. On the contrary, individuals with an entity mindset might give up further attempts under the pretext of a perceived ability deficiency when they experience processing disfluency. Overall, having an incremental mindset might serve as a buffer against the negative effects of the metacognitive disfluency experience on Chinese logogriphs solving.

Incremental mindset interventions have been popularized through multiple avenues (Blackwell et al. 2007; Yeager et al. 2019). Considering the significant positive correlations between individual’s creative mindset and their lifetime creative achievement, creative self-efficacy and creative problem-solving performance (Jia et al. 2022; Oconnor et al. 2013; Zhou et al. 2020), this concept provides a new perspective on improving individual’s creativity from their incremental creative mindset cultivation. Perhaps future creativity education could try to shape students’ incremental creative mindsets to facilitate their intrinsic creative motivation, beliefs, and achievement.

Several potential limitations of the present research should be noted. First, the effect of creative mindset manipulation on participants’ motivation should be tested. Just as the research on domain-general implicit theories of intelligence has found that participants with different implicit theories of intelligence may exhibit differences in task motivation, thereby influencing their final task performance (Dweck 1990), further studies are needed to clarify this possibility carefully. In addition, considering that creative mindsets have strong positive relationships with other creative self-concepts, such as creative self-efficacy and creative personal identity (Karwowski 2014), the role of creative mindsets in Chinese logogriphs solving might be partly influenced by these related variables. Further research is required to explore the influence of creative mindsets on creativity by exploring how these mindsets interact with related variables.

Second, other observed measurement indices are needed to directly reflect how individuals’ creative mindsets alter their interpretations of the metacognitive disfluency experience and subsequent Chinese logogriph task performance. According to the social-cognitive model of motivation and personality (Dweck and Leggett 1988), individuals could use their general theories of intelligence to form specific beliefs about effort and processing fluency; this difference in an individual’s beliefs on intelligence can alter their interpretation of effort and processing fluency which has been studied in a number of different domains such as student achievements, judgments of price, and reading comprehension (Thomas and Morwitz 2008; Miele et al. 2011). Similar to the study by Miele and Molden (2010). Self-paced study time, which is a spontaneous and naturalistic indicator of self-generated effort and cognitive persistence (Koriat and Ma’ayan 2005), was used in the present study to reflect how people with different theories of intelligence choose to allocate their study time in different processing fluency conditions. However, the limitation was that self-paced study time could be considered both a process variable of the time itself required to solve the logogriphs and a performance variable of the extent of effort and involved cognitive persistence. Therefore, other rigorous measurement indices should be used to further test the different processing fluency interpretations and employment efforts of individuals with different creative mindsets.

Third, the structure and measurement tools of the creative mindset should be reconsidered. In contrast to the present study, which assumed the incremental and entity mindsets to be two ends of the same continuum (Oconnor et al. 2013), Karwowski (2014) proposed that these two mindsets were negatively associated, distinct concepts that individuals could hold simultaneously. Considering the different ideas about the structure of the creative mindset, the measurement tools used to explore its relationship with creativity might vary. For example, along with the prime manipulation used to activate individuals’ creative mindsets in the present study, some psychometric scales, such as those developed by Dweck (1990) and Karwowski (2014), could be used in the future.

## 5. Conclusions

In summary, using the Chinese logogriph task, the present study showed that individuals with an incremental creative mindset had significantly higher performance accuracy and longer reaction times for logogriphs with a difficult font style than those with an entity creative mindset. These findings demonstrated that metacognitive disfluency experience impeded the Chinese logogriph problem solving, but incremental creative mindsets could mitigate this negative effect by cognitive persistence. This study provides novel evidence for the dual-process theory of creative thinking.

## Figures and Tables

**Table 1:** Means (standard deviations) of the difficulty for different kinds of logogriphs.

	Task Difficulty				
Font Style	Easy	Difficult	*t*	*p*	*d_z_*
Easy	0.63 (0.05)	0.94 (0.07)	−11.52	<0.001	3.62
Difficult	0.62 (0.05)	0.95 (0.07)	−12.44	<0.001	3.97

**Table 2:** Estimates of fixed effects on performance accuracy and insight value.

	Performance Accuracy				Insight Value			
Effects	*β*	*SE*	*t* Value	*p*	*β*	*SE*	*t* Value	*p*
Intercept	0.64	0.02	31.87	<0.001	3.14	0.2	16.13	<0.001
Task difficulty	−0.50	0.02	−20.99	<0.001	−0.50	0.2	−2.22	<0.05
Font style	−0.31	0.02	−13.03	<0.001	−0.24	0.2	−1.18	0.24
Task difficulty×Font style	0.27	0.03	8.17	<0.001	−0.07	0.29	−0.26	0.80

**Table 3:** Estimates of fixed effects on performance accuracy and reaction time.

	Performance Accuracy				Reaction Time			
Effects	*β*	*SE*	*t* Value	*p*	*β*	*SE*	*t* Value	*p*
Intercept	0.45	0.02	19.1	<0.001	7.15	0.41	17.53	<0.001
Creative mindset	0.02	0.03	0.47	0.64	−1.17	0.58	−2.03	<0.05
Font style	−0.08	0.03	−3.22	<0.005	1.00	0.58	1.73	0.09
Creative mindset × Font style	0.08	0.04	2.27	<0.05	2.57	0.82	3.15	<0.005

## Data Availability

The data are currently not publicly available due to participant privacy, but they are available from the first author upon reasonable request.

## References

[B1-jintelligence-11-00099] Aiello Daniel A., Jarosz Andrew F., Cushen Patrick J., Wiley Jennifer (2012). Firing the executive: When an analytic approach to problem solving helps and hurts. The Journal of Problem Solving.

[B2-jintelligence-11-00099] Alter Alter L., Oppenheimer Daniel M. (2009). Uniting the Tribes of Fluency to Form a Metacognitive Nation. Personality and Social Psychology Review.

[B3-jintelligence-11-00099] Alter Adam L., Oppenheimer Daniel M., Epley Nicholas, Eyre Rebecca N. (2007). Overcoming intuition: Metacognitive difficulty activates analytic reasoning. Journal of Experimental Psychology: General.

[B4-jintelligence-11-00099] Andiliou Andria, Murphy P. Karen (2010). Examining variations among researchers’ and teachers’ conceptualizations of creativity: A review and synthesis of contemporary research. Educational Research Review.

[B5-jintelligence-11-00099] Ansburg Pamela I., Hill Katherine (2003). Creative and analytic thinkers differ in their use of attentional resources. Personality and Individual Differences.

[B6-jintelligence-11-00099] Bates Douglas, Mächler Martin, Bolker Ben, Walker Steve (2015). Fitting Linear Mixed-Effects Models Using lme4. Journal of Statistical Software.

[B7-jintelligence-11-00099] Benedek Mathias, Bergner Sabine, Könen Tanja, Fink Andreas, Neubauer Aljoscha C. (2011). EEG alpha synchronization is related to top-down processing in convergent and divergent thinking. Neuropsychologia.

[B8-jintelligence-11-00099] Berkowitz Aaron L., Ansari Daniel (2008). Generation of novel motor sequences: The neural correlates of musical improvisation. Neuroimage.

[B9-jintelligence-11-00099] Blackwell Lisa S., Trzesniewski Kali H., Dweck Carol S. (2007). Implicit Theories of Intelligence Predict Achievement Across an Adolescent Transition: A Longitudinal Study and an Intervention. Child Development.

[B10-jintelligence-11-00099] Boksman Kristine, Théberge Jean, Williamson Peter C., Drost Dick J., Malla Ashok, Densmore Maria, Takhar Jatinder, Pavlosky William, Menon Ravi S., Neufeld Richard W. J. (2005). A 4.0-T fMRI study of brain connectivity during word fluency in first-episode schizophrenia. Schizophrenia Research.

[B11-jintelligence-11-00099] Botvinick Matthew M. Braver, Barch Todd S., Carter Deanna M., Cohen Cameron S., Jonathan D. (2001). Conflict monitoring and cognitive control. Psychological Review.

[B12-jintelligence-11-00099] Brand Serge, Reimer Torsten, Opwis Klaus (2007). How do we learn in a negative mood? Effects of a negative mood on transfer and learning. Learning and Instruction.

[B13-jintelligence-11-00099] Chen Qun, Luo Jun, Jiang Jun, Wei Dong, Zhang Qing (2012). Effects of Unconscious Processing on Creativity Problems Solving. Psychological Devclopment and Education.

[B14-jintelligence-11-00099] Chiu Chi, Ying Yue, Hong Yi, Dweck Carol S. (1997). Lay dispositionism and implicit theories of personality. Journal of Personality & Social Psychology.

[B15-jintelligence-11-00099] De Dreu Carsten K. W., Baas Matthijs, Nijstad Bernard A. (2008). Hedonic tone and activation level in the mood-creativity link: Toward a dual pathway to creativity model. Journal of Personality & Social Psychology.

[B16-jintelligence-11-00099] Dweck Carol S. (1990). Self-Theories: Their Role in Motivation, Personality, and Development. Essays in Social Psychology. Nebraska Symposium on Motivation.

[B17-jintelligence-11-00099] Dweck Carol S., Leggett Ellen L. (1988). A Social-Cognitive Approach to Motivation and Personality. Psychological Review.

[B18-jintelligence-11-00099] Erbas Ayhan Kursat, Bas Selda (2015). The Contribution of Personality Traits, Motivation, Academic Risk-Taking and Metacognition to the Creative Ability in Mathematics. Creativity Research Journal.

[B19-jintelligence-11-00099] Eysenck Hans. J. (1993). Creativity and Personality: Suggestions for a Theory. Psychological Inquiry.

[B20-jintelligence-11-00099] Faul Franz, Erdfelder Edgar, Lang Albert-Georg, Buchner Axel (2007). G*Power 3: A flexible statistical power analysis program for the social, behavioral, and biomedical sciences. Behavior Research Methods.

[B21-jintelligence-11-00099] Fink Andreas, Koschutnig Karl, Benedek Mathias, Reishofer Gernot, Ischebeck Anja, Weiss Elisabeth M., Ebner Franz (2012). Stimulating creativity via the exposure to other people’s ideas. Human Brain Mapping.

[B22-jintelligence-11-00099] Flavell John H., Resnick Lauren B. (1976). Metacognitive aspects of problem solving. The Nature of Intelligence.

[B23-jintelligence-11-00099] Förster Jens, Friedman Ronald S., Liberman Nira (2004). Temporal Construal Effects on Abstract and Concrete Thinking: Consequences for Insight and Creative Cognition. Journal of Personality and Social Psychology.

[B24-jintelligence-11-00099] Gabora Liane (2005). Creative Thought as a non-Darwinian Evolutionary Process. Journal of Creative Behavior.

[B25-jintelligence-11-00099] Gilhooly Kenneth J., Fioratou Evridiki, Anthony Susan H., Wynn V. (2007). Divergent thinking: Strategies and executive involvement in generating novel uses for familiar objects. British Journal of Psychology.

[B26-jintelligence-11-00099] Guilford Joy Paul (1967). Creativity: Yesterday, Today and Tomorrow. The Journal of Creative Behavior.

[B27-jintelligence-11-00099] Hong Ying, Chiu Chi, Dweck Carol S., Lin Derrick M. S., Wan Wendy (1999). Implicit theories, attributions, and coping: A meaning system approach. Journal of Personality and Social Psychology.

[B28-jintelligence-11-00099] Howardjones Paul A. (2002). A Dual-state Model of Creative Cognition for Supporting Strategies that Foster Creativity in the Classroom. International Journal of Technology and Design Education.

[B29-jintelligence-11-00099] Jaeger T. Florian (2008). Categorical Data Analysis: Away from ANOVAs (Transformation or Not) and towards Logit Mixed Models. Journal of Memory and Language.

[B30-jintelligence-11-00099] James William (2007). The Principles of Psychology.

[B31-jintelligence-11-00099] Jausovec Norbert, Runco Mark A. (1994). Metacognition in Creative Problem-Solving. Problem Finding, Problem Solving, and Creativity.

[B32-jintelligence-11-00099] Jia Xiao, Li Ping, Li Xin, Zhang Yu, Cao Wei, Cao Li, Li Wei (2016). The Effect of Word Frequency on Judgments of Learning: Contributions of Beliefs and Processing Fluency. Frontiers in Psychology.

[B33-jintelligence-11-00099] Jia Xiao, Xu Tian, Zhang Yu (2022). The Role of Metacognitive Strategy Monitoring and Control in the Relationship between Creative Mindsets and Divergent Thinking Performance. Journal of Intelligence.

[B34-jintelligence-11-00099] Jia Xiao, Li Wei, Cao Li (2019). The Role of Metacognitive Components in Creative Thinking. Frontiers in Psychology.

[B35-jintelligence-11-00099] Jungbeeman Mark, Bowden Edward M., Haberman Jason, Frymiare Jennifer L., Arambel-Liu Stella, Greenblatt Richard, Reber Paul J., Kounios John (2004). Neural Activity When People Solve Verbal Problems with Insight. PLoS Biology.

[B36-jintelligence-11-00099] Karwowski Maciej (2014). Creative mindsets: Measurement, correlates, consequences. Psychology of Aesthetics, Creativity, and the Arts.

[B37-jintelligence-11-00099] Koriat Asher (2008). Easy comes, easy goes? The link between learning and remembering and its exploitation in metacognition. Memory & Cognition.

[B38-jintelligence-11-00099] Koriat Asher, Ma’ayan Hilit (2005). The Effects of Encoding Fluency and Retrieval Fluency on Judgments of Learning. Journal of Memory and Language.

[B39-jintelligence-11-00099] Koriat Asher, Bjork Robert A., Sheffer Limor, Bar Sarah K. (2004). Predicting One’s Own Forgetting: The Role of Experience-Based and Theory-Based Processes. Journal of Experimental Psychology: General.

[B40-jintelligence-11-00099] Kounios John, Fleck Jessica I., Green Deborah L., Payne Lisa, Stevenson Jennifer L., Bowden Edward M., Jung-Beeman Mark (2008). The origins of insight in resting-state brain activity. Neuropsychologia.

[B41-jintelligence-11-00099] Kühl Tim Alexander Eitel, Scheiter Katharina, Gerjets Peter (2014). A Call for an Unbiased Search for Moderators in Disfluency Research: Reply to Oppenheimer and Alter. Applied Cognitive Psychology.

[B42-jintelligence-11-00099] Labroo Aparna A., Kim Sara (2009). The “instrumentality” heuristic: Why metacognitive difficulty is desirable during goal pursuit. Psychological Science.

[B43-jintelligence-11-00099] Li Ping, Jia Xiao, Li Xin, Li Wei (2016). The effect of animacy on metamemory. Memory & Cognition.

[B44-jintelligence-11-00099] Lieberman Matthew D., Gaunt Ruth, Gilbert Daniel T. (2002). Reflexion and reflection: A social cognitive neuroscience approach to attributional inference. Advances in Experimental Social Psychology.

[B45-jintelligence-11-00099] Lizarraga María Luisa Sanz de Acedo, Baquedano María Teresa Sanz de Acedo (2013). How creative potential is related to metacognition. European Journal of Education and Psychology.

[B46-jintelligence-11-00099] Lucas Brian J., Nordgren Loran F. (2015). People underestimate the value of persistence for creative performance. Journal of Personality and Social Psychology.

[B47-jintelligence-11-00099] Luo Jun, Knoblich Guenther (2007). Studying insight problem solving with neuroscientific methods. Methods.

[B48-jintelligence-11-00099] Luo Jun, Li Wen, Fink Andreas, Jia Lei, Xiao Xiao, Qiu Jiang, Zhang Qing (2011). The time course of breaking mental sets and forming novel associations in insight-like problem solving: An ERP investigation. Experimental Brain Research.

[B49-jintelligence-11-00099] Mehta Ravi P., Zhu Rui, Cheema Amar (2012). Is Noise Always Bad? Exploring the Effects of Ambient Noise on Creative Cognition. Journal of Consumer Research.

[B50-jintelligence-11-00099] Metcalfe Janet, Wiebe David (1987). Intuition in insight and noninsight problem solving. Memory & Cognition.

[B51-jintelligence-11-00099] Miele David B., Molden Daniel C. (2010). Naive theories of intelligence and the role of processing fluency in perceived comprehension. Journal of Experimental Psychology: General.

[B52-jintelligence-11-00099] Miele David B., Finn Bridgid, Molden Daniel C. (2011). Does Easily Learned Mean Easily Remembered? It Depends on Your Beliefs About Intelligence. Psychological Science.

[B53-jintelligence-11-00099] Miele David B., Son Lisa K., Metcalfe Janet (2013). Children’s Naive Theories of Intelligence Influence Their Metacognitive Judgments. Child Development.

[B54-jintelligence-11-00099] Molden Daniel C., Dweck Carol S. (2006). Finding “meaning” in psychology: A lay theories approach to self-regulation, social perception, and social development. American Psychologist.

[B55-jintelligence-11-00099] Nijstad Bernard A., De Dreu Carsten K. W., Rietzschel Eric F., Baas Matthijs (2010). The dual pathway to creativity model: Creative ideation as a function of flexibility and persistence. European Review of Social Psychology.

[B56-jintelligence-11-00099] Novemsky Nathan, Dhar Ravi, Schwarz Norbert, Simonson Itamar (2007). Preference fluency in choice. Journal of Marketing Research.

[B57-jintelligence-11-00099] Oconnor Alexander J., Nemeth Charlan J., Akutsu Satoshi (2013). Consequences of Beliefs about the Malleability of Creativity. Creativity Research Journal.

[B58-jintelligence-11-00099] Preiss David D., Cosmelli Diego, Grau Valeska, Ortiz Dominga (2016). Examining the influence of mind wandering and metacognition on creativity in university and vocational students. Learning and Individual Differences.

[B59-jintelligence-11-00099] Puente-Díaz Rogelio (2023). Metacognitive Feelings as a Source of Information for the Creative Process: A Conceptual Exploration. Journal of Intelligence.

[B60-jintelligence-11-00099] Qiu Jiang, Li Hong, Yang Dong, Luo Yue, Li Ying, Wu Zhen, Zhang Qing (2008). The neural basis of insight problem solving: An event-related potential study. Brain and Cognition.

[B61-jintelligence-11-00099] Qiu Jiang, Li Hong, Jou Jerwen, Liu Jia, Luo Yue, Feng Ting, Wu Zhen, Zhang Qing (2010). Neural correlates of the “Aha” experiences: Evidence from an fMRI study of insight problem solving. Cortex.

[B62-jintelligence-11-00099] Reber Rolf, Wurtz Pascal, Zimmermann Thomas D. (2004). Exploring “fringe” consciousness: The subjective experience of perceptual fluency and its objective bases. Consciousness and Cognition.

[B63-jintelligence-11-00099] Roskes Marieke, De Dreu Carsten K. W., Nijstad Bernard A. (2012). Necessity Is the Mother of Invention: Avoidance Motivation Stimulates Creativity Through Cognitive Effort. Journal of Personality and Social Psychology.

[B64-jintelligence-11-00099] Rubensteina Lisa DaVia, Callanb Gregory L., Ridgleya Lisa M., Henderson Amanda (2019). Students’ strategic planning and strategy use during creative problem solving: The importance of perspective-taking. Thinking Skills and Creativity.

[B65-jintelligence-11-00099] Skaar Øystein O., Reber Rolf (2020). The phenomenology of Aha-experiences. Motivation Science.

[B66-jintelligence-11-00099] Sowden Paul T., Pringle Andrew, Gabora Liane (2015). The shifting sands of creative thinking: Connections to dual-process theory. Thinking & Reasoning.

[B67-jintelligence-11-00099] Sprugnoli Giulia, Rossi Simone, Emmendorfer Alexandra, Rossi Alessandro, Liew Sook-Lei, Tatti Elisa, di Lorenzo Giorgio, Pascual-Leone Alvaro, Santarnecchi Emiliano (2017). Neural correlates of Eureka moment. Intelligence.

[B68-jintelligence-11-00099] Sternberg Robert J., Lubart Todd I. (1991). An Investment Theory of Creativity and Its Development. Human Development.

[B69-jintelligence-11-00099] Storbeck Justin, Clore Gerald L. (2007). On the interdependence of cognition and emotion. Cognition & Emotion.

[B70-jintelligence-11-00099] Thomas Manoj, Morwitz Vicki G. (2008). The Ease-of-Computation Effect: The Interplay of Metacognitive Experiences and Naive Theories in Judgments of Price Differences. Journal of Marketing Research.

[B71-jintelligence-11-00099] Threadgold Emma, Marsh John E., McLatchie Neil, Ball Linden J. (2019). Background music stints creativity: Evidence from compound remote associate tasks. Applied Cognitive Psychology.

[B72-jintelligence-11-00099] Topolinski Sascha, Reber Rolf (2010). Gaining Insight Into the “Aha” Experience. Current Directions in Psychological Science.

[B73-jintelligence-11-00099] West Keon, Bruckmüller Susanne (2013). Nice and easy does it: How perceptual fluency moderates the effectiveness of imagined contact. Journal of Experimental Social Psychology.

[B74-jintelligence-11-00099] Wu Zhen, Jiang Qiu, Zhang Qing (2009). Exploring a New Experimental Paradigm in the Brain Research of Insight. Psychological Science.

[B75-jintelligence-11-00099] Yeager David S., Hanselman Paul, Walton Gregory M., Murray Jared S., Crosnoe Robert, Muller Chandra, Tipton Elizabeth, Schneider Barbara, Hulleman Chris S., Hinojosa Cintia P. (2019). A national experiment reveals where a growth mindset improves achievement. Nature.

[B76-jintelligence-11-00099] Zhou Yi, Yang Wa, Bai Xin (2020). Creative mindsets: Scale validation in the Chinese setting and generalization to the real workplace. Frontiers in Psychology.

